# Metabolomic analysis identifies alterations of amino acids in the tears and plasma of patients with dry eye disease with ocular pain

**DOI:** 10.3389/fmed.2025.1733856

**Published:** 2026-01-09

**Authors:** Lan Ke, Wanju Yang, Kuiliang Yang, Jiewen Mao, Yujin Wang, Dongping Li, Yiqiao Xing, Qingyan Zeng, Yanning Yang

**Affiliations:** 1 Department of Ophthalmology, Renmin Hospital of Wuhan University, Wuhan, Hubei, China; 2 Aier Eye Hospital of Wuhan University, Wuhan, Hubei, China; 3 Wuhan Aier Hankou Eye Hospital, Wuhan, Hubei, China

**Keywords:** amino acid, corneal nerves, dry eye, metabolomic analysis, ocular pain

## Abstract

**Objectives:**

Identifying the amino acid in tears and plasma and investigating the correlation between amino acid concentrations and the presence of dry eye disease (DED) with ocular pain.

**Methods:**

15 participants in the DED with ocular pain group, 19 in the DED group, and 16 in the control group were enrolled and underwent DED examinations. Evaluation parameters included the Ocular Surface Disease Index score, Numeric Rating Scale score, clinical ocular parameters, as well as measurements from *in vivo* laser confocal microscopy. Amino acid concentrations were analyzed using the Waters ACQUITY UPLC I-Class/Xevo TQ-S micro system.

**Results:**

A total of 29 and 36 distinct amino acids were identified in tear fluid and plasma, respectively. The results showed significantly higher levels of methionine (MET) in tear fluid and 1-methyl-L-histidine (1-MEHIS) in plasma in people who developed DED with ocular pain compared to those who did not (*p* = 0.003, *q*-value = 0.044; *p* < 0.001, *q*-value < 0.001), with receiver-operating characteristic analysis yielding an AUC of 0.686 and 0.869.

**Conclusion:**

MET in tear fluid and 1-MEHIS in plasma were significantly associated with DED with ocular pain, showing promise as potential biomarkers for DED with ocular pain. Further validation through large-scale studies is warranted.

## 1 Introduction

Dry eye disease (DED) is a chronic ocular surface disorder influenced by multifactorial etiologies, often accompanied by inflammatory responses, tissue damage, and neural abnormalities, which collectively contribute to a spectrum of uncomfortable ocular symptoms and visual dysfunctions (1). With the increasing prevalence of digital device usage, worsening air pollution, and the progression of population aging, the incidence of DED has been steadily rising. Current data suggest an annual increase in incidence rates ranging from approximately 5 to 50% (2). Clinical manifestations of DED often include sensations such as foreign body presence, burning, and prickling discomfort, and ocular pain affects 89% of patients with DED, significantly impairing their quality of life and contributing to a substantial economic burden on society (3).

In recent years, there has been growing clinical interest in understanding the role of ocular pain in DED. Literature suggests that DED with ocular pain is potentially associated with unstable tear film, abnormal tear secretion, ocular surface inflammation, and reduced corneal nerve density (4–6). However, the precise pathophysiological mechanisms remain unclear and treatment strategies for DED with ocular pain remain limited. Further in-depth investigations are critical for developing targeted therapeutic approaches.

Amino acids, as essential nutrients and regulators of various metabolic processes, play a fundamental role in maintaining cellular homeostasis. Changes in amino acid concentrations in body fluids offer a promising avenue for the development of diagnostic and prognostic biomarkers. Specifically, tear fluid markers have demonstrated potential for early diagnosis and monitoring of ocular disease progression (7–9). Previous studies employing mass spectrometry have revealed that DED pathogenesis is primarily driven by immune and inflammatory mechanisms. Key metabolic pathways, including complement and coagulation cascades, glycolysis/gluconeogenesis, and amino acid metabolism, have been implicated in the disease process (10–12). Alterations in amino acid metabolism are known to modulate oxidative stress on the ocular surface, exacerbating inflammation and contributing to tissue damage in affected individuals (9). Despite these findings, there is a notable gap in research addressing whether specific amino acids in the tears or blood of patients with DED with ocular pain might influence disease onset, progression, or diagnostic outcomes.

This study primarily detects amino acids in the tears and plasma of patients with DED experiencing ocular pain to investigate potential amino acids associated with the condition.

## 2 Participants and methods

### 2.1 Participants and study design

This study employed a cross-sectional, observational design conducted during a single visit, with participants stratified into three groups: the DED with ocular pain group, the DED group, and the control group. Both the left and right eyes of participants were randomly selected for inclusion. Data collection was comprehensive, encompassing demographic information, the Ocular Surface Disease Index (OSDI) score, Numeric Rating Scale (NRS) scores, clinical ocular indicators, as well as the collection of tear fluid and plasma samples. Additionally, *in vivo* laser confocal microscopy (IVCM) examinations were performed. Subsequently, amino acid concentrations in both tear fluid and plasma were quantified.

The diagnostic criteria for DED with ocular pain were as follows (6): OSDI ≥ 13 points, Schirmer I < 10 mm/5 min, tear film breakup time (FBUT) ≤ 7 s, and NRS ≥ 2 points. The diagnostic criteria for DED: OSDI ≥ 13 points, Schirmer I < 10 mm/5 min, FBUT ≤ 7 s, and NRS < 2 points.

Inclusion criteria were as follows: (1) Voluntary participation in the study, with signed informed consent; (2) Age between 18 and 70 years; (3) No history of contact lens use within the past 6 month; (4) No participation in other clinical trials within the past month; (5) No current use of eye or systemic medications, or if used, cessation for more than one month prior to the study.

Exclusion criteria were as follows: (1) Allergy to any component of the test reagents; (2) Active ocular surface infection or recent ocular surface infection (within the past month); (3) Ocular surgery or trauma within the past 6 month; (4) Severe systemic diseases, including those affecting the cardiovascular, cerebrovascular, liver, kidney, thyroid, or hematopoietic systems; (5) Recent coronary stent implantation or history of coronary artery disease; (6) Diagnosed glaucoma or diabetic retinopathy; (7) Use of vitamin and/or folic acid supplements; (8) Pregnant or lactating women; (9) Any individual deemed unsuitable for participation by the researcher, such as those with mental disorders, substance abuse history, or poor compliance during exams.

The study was approved by the Ethics Committee of Wuhan Aier Hankou Eye Hospital (approval number: HKAIER2023IRB-09-01) and was conducted in accordance with the Declaration of Helsinki. Additionally, the study is registered with the Chinese Clinical Trial Registry (registration number: ChiCTR230007634). All participants provided written informed consent prior to inclusion in the study.

### 2.2 Methods

#### 2.2.1 Basic information

The collected data encompassed the following variables: participant name, gender, age (in years), race, history of drug allergies, past medical and surgical histories, duration of DED (in months) and average daily sleep duration (in hours).

#### 2.2.2 The OSDI and NRS score

The OSDI score (13) consists of three sections. The first section evaluates ocular symptoms (questions 1–5, with a scoring range of 0–20). The second section assesses the impact of ocular discomfort on daily life and work (questions 6–9, with a scoring range of 0–16). The third section examines the contribution of environmental factors to ocular discomfort (questions 10–12, with a scoring range of 0–12). The total score, which ranges from 0 to 100, is calculated by the formula: (Total Score / Number of Questions Answered) × 25.

The NRS (14) is represented as a 10-cm visual analog scale, divided into 100 equal graduations. The endpoints are designated as “0” (no pain, marked with a smiling face) and “10” (intolerably severe pain, marked with a crying face, representing pain so extreme it would necessitate the removal of the eyeball). Participants are asked to mark the point on the scale that corresponds to their pain level, with the total score ranging from 0 to 10.

#### 2.2.3 Ocular examination

##### 2.2.3.1 Corrected visual acuity

Participants stood 5 meters away from the standard logarithmic visual acuity chart and read the visual targets sequentially from top to bottom until they could no longer discern them clearly. Corrected visual acuity was recorded.

##### 2.2.3.2 Intraocular pressure (IOP)

IOP for each eye was assessed using a non-contact tonometer (NT-510, NIDEK, Japan), with the average value calculated from three separate measurements.

##### 2.2.3.3 FBUT

The time interval between blinking and the first tear film rupture was measured three times, with the average value used for analysis. FBUT ≤ 7 s was considered abnormal (15).

##### 2.2.3.4 Schirmer I test

A Schirmer test strip (Liaoning Meizilin Pharmaceutical Co., Ltd., China) was placed in the outer one-third of the lower temporal conjunctival fornix without topical anesthetics. Participants were instructed to gently close their eyes and refrain from moving their eyeballs. After 5 min, the length of the moistened portion of the strip was measured in millimeters (mm) (15).

##### 2.2.3.5 National Eye Institute (NEI) score

The Oxford grading system (0–5 areas) was used to assess the integrity of the ocular surface following corneal fluorescein staining (Tianjin Jingming New Technology Development Co., Ltd., China), with each of the five areas receiving a score between 0 and 3, resulting in a total score ranging from 0 to 15. A higher score indicates a greater degree of ocular surface abnormality (15, 16).

#### 2.2.4 Preparation of tear fluids samples

Fifty microliters of 0.9% sodium chloride injection (Tangpa Medical Co., Ltd., China) were administered into the lower conjunctival fornix using a pipette (17). Participants were instructed to gently close their eyes and tilt their heads to one side. Tear fluid flushed from the conjunctival fornix was collected using a 20 μL microcapillary (Jiangsu Kangjianhua Medical Co., Ltd., China) within a 1-min collection window. The collected tear fluid was transferred from the microcapillary to a 100 μL centrifuge tube (Wuhan Servicebio Technology Co., Ltd., China), then centrifuged, and stored at −80 °C for subsequent analysis. All participants’ tear samples were collected by the same examiner on the second day of enrollment, between 8:00 and 11:00 daily in the same indoor room, with the room temperature controlled at approximately 20 °C and the relative humidity maintained at around 35%.

#### 2.2.5 Preparation of plasma samples

Participants refrained from food and fluid intake for 8 h prior to sample collection. Peripheral venous blood was drawn into an anticoagulant tube (HuBei JinXing Technology Development Co., Ltd., China), inverted gently, and centrifuged at 2750 × g using a centrifuge (320A, Beijing Baiyang Medical Equipment Co., Ltd.) for 10 min at room temperature (18). The resulting supernatant was transferred to a pre-chilled 100 μL centrifuge tube (Beijing Labgic Technology Co., Ltd., China) using a pipette, vortexed for 1 min (Haimen Kylin-Bell Lab Instruments Co., Ltd., China), and stored at −80 °C until analysis. The entire process, from blood collection to vortexing, was completed within 30 min.

#### 2.2.6 IVCM and image analysis

The corneal subbasal nerve plexus was examined using *in vivo* confocal microscopy (IVCM) (Heidelberg Engineering, GmBH, Heidelberg, Germany) in the central cornea and at four quadrants approximately 3 mm from the center of each participant’s eye (19). For each orientation of the eye, five images were selected (a total of 25 images per eye). Only images with a nerve coincidence rate not exceeding 1/10 were included for analysis. The 25 images from each eye were grouped into a single folder, and the ACC Metrics software (The University of Manchester, United Kingdom) was employed to analyze various parameters for each image within the folder. The analyzed parameters included: Corneal Nerve Fiber Density (CNFD) (number of fibers per mm^2^), Corneal Nerve Branch Density (CNBD) (number of branch points on the main fibers per mm^2^), Corneal Nerve Fiber Length (CNFL) (total length of nerves in mm per mm^2^), Corneal Total Branch Density (CTBD) (total number of branch points per mm^2^), Corneal Nerve Fiber Area (CNFA) (total nerve fiber area in mm^2^ per mm^2^), Corneal Nerve Fiber Width (CNFW) (average nerve fiber width in mm per mm^2^), and Corneal Nerve Fiber Fractal Dimension (CNFracDim) (spatial loss of nerves). The average values for all parameters derived from the 25 images of each eye were subsequently calculated and used for further analysis.

#### 2.2.7 Detection of amino acid profiles in tears and plasma by the Waters ACQUITY UPLC I-Class/Xevo TQ-S micro system (Waters Corporation)

The processed plasma, tear fluid samples, and standards (Supplementary material) were subjected to chromatographic separation using a Waters ACQUITY UPLC I-CLASS chromatography system (chromatographic column: Waters UPLC HSS T3 (1.8 μm, 2.1 mm × 150 mm); mobile phase: Phase A [water (W6-4, Fisher Scientific, Waltham, MA), 0.1% formic acid (A117-50, Fisher Scientific, Waltham, MA)], Phase B [acetonitrile (A955-4, Fisher Scientific, Waltham, MA)]; elution gradient: Supplementary Table S1; flow rate: 0.5 mL/min; injection volume: 5.0 μL; column temperature: 50 °C). Mass spectrometry analysis was subsequently performed using a tandem quadrupole mass spectrometer (XEVO TQ-S Micro, Waters Corp, Milford, MA, USA) (ion source voltage 1.5 kV, cone voltage 20 V; desolvation temperature 600 °C, desolvation gas flow rate 1,000 L/h; cone gas flow rate 10 L/h). The peak areas were then integrated using Masslynx software for mass spectrometry data collection (TargetLynx, Waters Corp, Milford, MA, USA), with a retention time tolerance of 15 s. The final concentrations were calculated using the standard curve method (20).

Thirty-eight amino acids were detected in both plasma and tears: L-histidine (HIS), 1-methyl-L-histidine (1-MEHIS), 3-MEHIS, L-hydroxyproline (OHPRO), L-carnosine (CAR), L-arginine (ARG), L-asparagine (ASN), O-phosphoethanolamine (PHETH), L-anserine (Ans), taurine (TAU), L-glutamine (GLN), L-serine (SER), ethanolamine (ETH), glycine (GLY), L-aspartic acid (ASP), L-citrulline (CIT), L-sarcosine (SAR), L-glutamic acid (GLU), *β*-alanine (beta-ALA), L-threonine (THR), L-alanine (ALA), *γ*-aminobutyric acid (gamma-GABA), *δ*-D, L-hydroxylysine (OH-LYS), L-*α*-aminoadipic acid (AMADP), L-proline (PRO), D, L-β-aminoisobutyric acid (beta-GABA), L-ornithine (ORN), L-lysine (LYS), L-α-aminobutyric acid (alpha-GABA), L-cystine (CYS), cystathionine (CYST), L-tyrosine (TYR), L-methionine (MET), L-valine (VAL), L-isoleucine (ILE), L-leucine (LEU), L-phenylalanine (PHE), and L-tryptophan (TRP).

#### 2.2.8 Statistical analysis

Sample size calculation was performed based on previous literature (21). The following parameters were used: test power (1-*β*) = 0.9, significance level (*α*) = 0.05 (two-sided test), number of groups = 3, group allocation ratio = equal, and means multiplier = 1. The means ± *σ* values for the 23 amino acids detected were inputted into the calculation. The sample size was determined using PASS 15 software, yielding a total required sample size of 42 participants, with 14 participants per group, achieving a test power of 0.9204.

Data analysis was conducted using IBM SPSS Statistics 26.0. Missing values were imputed using multiple imputation. Quantitative variables with a normal distribution were presented as means ± standard deviation ( $\bar{x}\pm s$ ), with intergroup comparisons performed using Analysis of Variance (ANOVA). Post-hoc pairwise comparisons were conducted using the Student–Newman–Keuls (SNK) method. For quantitative variables that did not conform to a normal distribution, data were expressed as [M (IQR)], and intergroup comparisons were performed using the rank sum test, followed by post-hoc pairwise comparisons with the Bonferroni correction method. Qualitative data were expressed as counts (%), with intergroup comparisons made using Fisher’s exact probability test. Pearson correlation was used to analyze relationships between normally distributed quantitative variables, while Spearman correlation was applied for associations involving non-normally distributed or qualitative variables. False Discovery Rate (FDR) correction was used to account for the multiple amino acids tested. Receiver operating characteristic (ROC) curve analysis were used to assess the predictive ability of MET in tear fluid and 1-MEHIS in plasma, with cut-off values determined by the Youden Index. A *p*-value < 0.05 was considered statistically significant.

## 3 Results

### 3.1 Demographics

A total of 50 participants (50 eyes) were enrolled in this study, comprising 15 individuals with DED with ocular pain, 19 with DED, and 16 in the control group (Table 1). The gender distribution was 24% male and 76% female, with an average age of 32.38 ± 8.14 years. The average duration of DED was 24.00 (0.00, 45.00) months, and the average sleep duration was 7.00 (7.00, 8.00) hours per day. No significant difference was observed between the DED with ocular pain [36.00 (12.00, 64.00) months] and the DED groups [24.00 (24.00, 60.00) months] (*H* = 33.409, *p* < 0.001) in the duration of DED.

**Table 1:** Demographics of participants.

Variable	Total	DED with ocular pain (*n* = 15)	DED (*n* = 19)	Control (*n* = 16)	*F*/*H*	*p*
Gender						
Male	12 (24.00)	1 (6.67)	6 (31.58)	5 (31.25)		0.169*
Female	38 (76.00)	14 (93.33)	13 (68.42)	11 (68.75)		
Age (in years)	32.38 ± 8.14	34.27 ± 11.49	33.16 ± 6.83	29.69 ± 5.04	*F* = 1.385	0.260
Race						
Han	50 (100.00)	15 (100.00)	19 (100.00)	16 (100.00)		
Drug allergy history						
No	44 (88.00)	12 (80.00)	18 (94.74)	14 (87.50)		0.415*
Yes	6 (12.00)	3 (20.00)	1 (5.26)	2 (12.50)		
Previous history						
No	35 (70.00)	8 (53.33)	15 (78.95)	12 (75.00)		0.292*
Yes	15 (30.00)	7 (46.67)	4 (21.05)	4 (25.00)		
Surgical history						
No	37 (74.00)	8 (53.33)	16 (84.21)	13 (81.25)		0.121*
Yes	13 (26.00)	7 (46.67)	3 (15.79)	3 (18.75)		
Duration of DED (in months)	24.00 (0.00, 45.00)	36.00 (12.00, 64.00)	24.00 (24.00, 60.00)	0.00 (0.00, 0.00)^ab^	*H* = 33.409	<0.001^#^
Average sleep time per day (in hours)	7.00 (7.00, 8.00)	7.00 (6.50, 8.00)	7.00 (7.00, 7.50)	7.50 (7.00, 8.00)	*H* = 2.970	0.226^#^

*Fisher’s exact probability method; ^#^the rank sum test; ^a^indicates that there was a statistically significant difference between the DED with ocular pain group after pairwise comparison; ^b^indicates that the difference was statistically significant compared to the DED group after pairwise comparison.

### 3.2 Ocular examination and the OSDI and NRS scores of participants

The FBUT [2.00 (2.00, 3.00)] in the DED group was significantly lower than that in the DED with ocular pain group [3.00 (2.00, 3.00)], and the NEI score [1.00 (0.00, 3.00)] in the DED group was significantly higher than that in the control group [0.00 (0.00, 0.50)], with significant differences (all *p* < 0.05). The NRS score was significantly negatively correlated with Schirmer I test (*r* = −0.329, *p* < 0.05), while it was positively correlated with OSDI score (*r* = 0.446, *p* = 0.001), but not significantly correlated with NEI score and FBUT (both *p* > 0.05) (Table 2).

**Table 2:** Ocular examination and the OSDI and NRS scores of participants.

Variable	Total	DED with ocular pain (*n* = 15)	DED (*n* = 19)	Control (*n* = 16)	*F*/*H*	*p*
Corrected visual acuity	1.00 (1.00, 1.00)	1.00 (1.00, 1.00)	1.00 (1.00,1.00)	1.00 (1.00, 1.00)	*H* = 0.296	0.862#
IOP (mmHg)	14.60 ± 2.51	14.33 ± 2.69	15.05 ± 2.46	14.31 ± 2.47	*F* = 0.489	0.616
FBUT (sec)	3.00 (2.00, 5.00)	3.00 (2.00, 3.00)	2.00 (2.00, 3.00)^a^	7.83 (4.50, 10.00)^ab^	*H* = 17.713	<0.001#
Schirmer I test (mm/5 min)	7.00 (4.00, 20.00)	5.00 (3.00, 8.00)	5.00 (3.00, 7.00)	26.00 (20.00, 29.00)^ab^	*H* = 25.340	<0.001#
NEI score (0–15 points)	1.00 (0.00, 2.00)	1.00 (0.00, 2.00)	1.00 (0.00, 3.00)	0.00 (0.00, 0.50)^b^	*H* = 9.788	0.007#
OSDI score (points)	20.00 (11.36, 29.37)	27.08 (22.50, 40.00)	20.00 (15.90, 25.00)	5.68 (2.60, 11.36)^ab^	*H* = 23.145	<0.001#
NRS score (0–10 points)	0.40 (0.00, 3.00)	6.00 (3.00, 7.00)	0.00 (0.00, 0.80)^a^	0.00 (0.00, 0.15)^a^	*H* = 34.335	<0.001#

^#^The rank sum test; ^a^indicates that there was a statistically significant difference between the DED with ocular pain group after pairwise comparison; ^b^indicates that the difference was statistically significant compared to the DED group after pairwise comparison.

DED, dry eye disease; IOP, intraocular pressure; FBUT, tear film breakup time; NEI score, the National Eye Institute; OSDI, the Ocular Surface Disease Index; NRS score, the Numeric Rating Scale; mmHg, millimeter of mercury; sec, seconds; mm, millimeter; min, minutes.

### 3.3 IVCM parameters in participants

**Figure 1:**
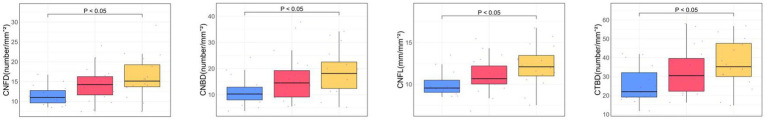
Comparison of the CNFD, CNBD, CNFL, CTBD in the DED with ocular pain group (blue), the DED group (red), and the control group (yellow).

Compared to the control group, both the DED with ocular pain and DED groups exhibited reductions in IVCM parameters (Table 3). Specifically, the DED with ocular pain group demonstrated significantly lower values in CNFD (11.43 ± 2.42), CNBD (11.30 ± 5.60), CNFL (10.00 ± 1.46), and CTBD (25.72 ± 9.75) compared to the control group (CNFD: 16.12 ± 5.39, CNBD: 18.87 ± 8.62, CNFL: 12.23 ± 2.50, CTBD: 36.73 ± 12.74), with all comparisons showing statistical significance (all *p* < 0.05) (Figure 1).

**Table 3:** IVCM parameters of participants.

IVCM parameters	Total	DED with ocular pain (*n* = 15)	DED (*n* = 19)	Control (*n* = 16)	*F*/*H*	*p*
CNFD (number/mm^2^)	13.99 ± 4.53	11.43 ± 2.42	14.21 ± 4.19	16.12 ± 5.39^a^	*F* = 4.841	0.012
CNBD (number/mm^2^)	15.51 ± 8.48	11.30 ± 5.60	16.00 ± 9.19	18.87 ± 8.62^a^	*F* = 3.453	0.039
CNFL (mm/mm^2^)	11.11 ± 2.21	10.00 ± 1.46	11.06 ± 2.08	12.23 ± 2.50^a^	*F* = 4.506	0.016
CTBD (number/mm^2^)	31.85 ± 12.48	25.72 ± 9.75	32.57 ± 12.72	36.73 ± 12.74^a^	*F* = 3.363	0.043
CNFA (mm/mm^2^)	0.01 ± 0.00	0.00 ± 0.00	0.01 ± 0.00	0.01 ± 0.00	*F* = 2.853	0.068
CNFW (mm/mm^2^)	0.02 ± 0.00	0.02 ± 0.00	0.02 ± 0.00	0.02 ± 0.00	*F* = 1.128	0.332
CNFracDim	1.45 ± 0.03	1.44 ± 0.02	1.45 ± 0.03	1.46 ± 0.03	*F* = 2.824	0.069

^a^Indicates that there was a statistically significant difference between the DED with ocular pain group after pairwise comparison.

IVCM, laser *in vivo* confocal microscopy; DED, dry eye disease; CNFD, corneal nerve fiber density; CNBD, corneal nerve branch density; CNFL, corneal nerve fiber length; CTBD, corneal total branch density; CNFA, corneal nerve fiber area; CNFW, corneal nerve fiber width; CNFracDim, corneal nerve fiber fractal dimension; mm^2^, square millimeter; mm, millimeter.

### 3.4 The detection results of amino acids in tear fluid

**Figure 2:**
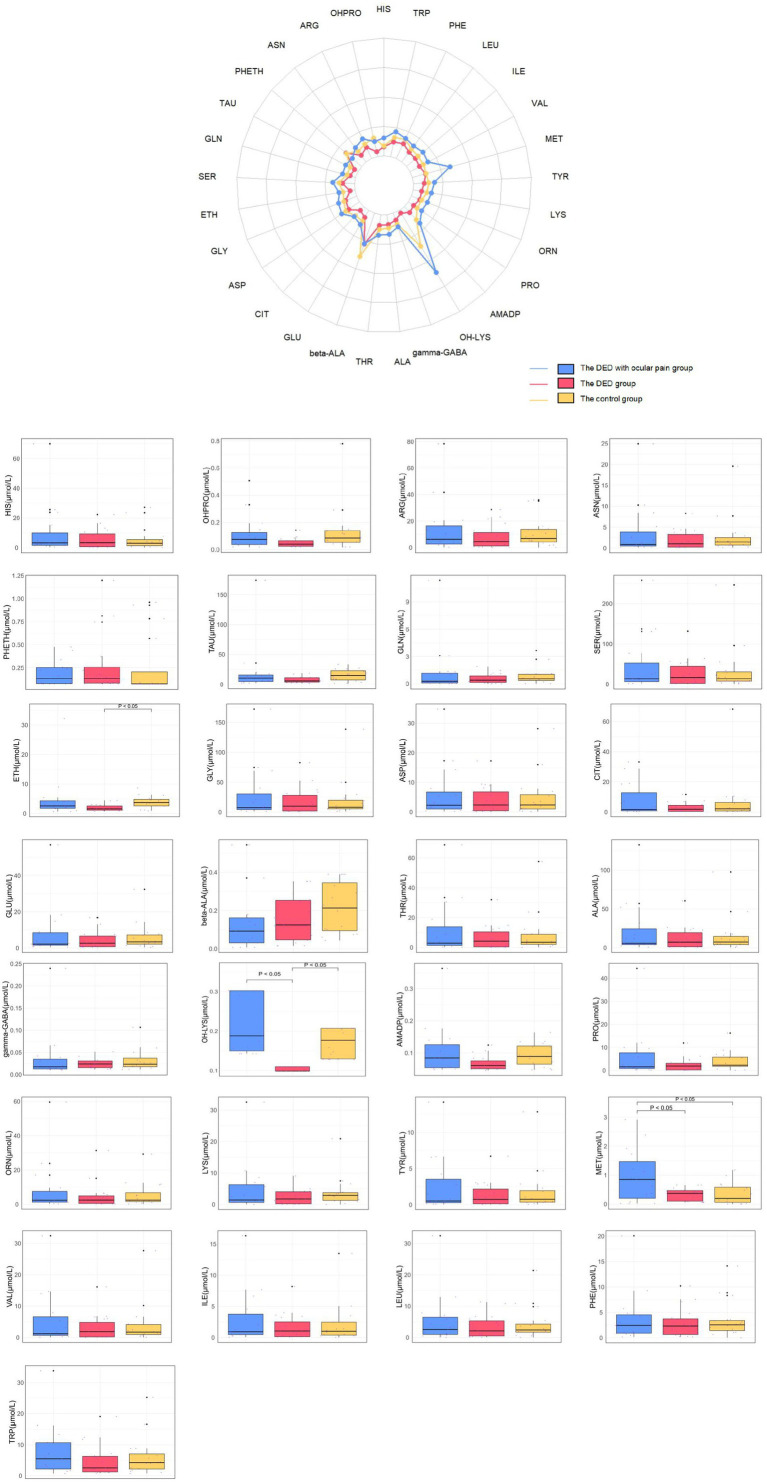
Differences in amino acid concentrations in tear fluid between the three groups. Top: Means values of 29 amino acid concentrations (μmol/L) of tear fluid of three groups are shown in the radar chart. Bottom: The average concentration of amino acids in tear fluid in three groups are shown in the box plots.

A total of 29 amino acids were detected in the tear fluid (Table 4; Figure 2). Significant differences were noted in the levels of ETH (*H* = 8.785, *p* = 0.012), OH-LYS (*H* = 36.684, *p* < 0.001), and MET (*F* = 6.396, *p* = 0.003) across the groups. The MET concentration in the DED with ocular pain group (0.97 ± 0.92) was significantly higher than that in both the DED group (0.34 ± 0.22) and the control group (0.36 ± 0.37) (all *p* < 0.05). In contrast, OH-LYS levels in the DED group [0.10 (0.10, 0.11)] were lower than those observed in the other two groups [DED with ocular pain group: 0.19 (0.14, 0.30); control group: 0.18 (0.13, 0.21)], and ETH levels [1.58 (1.07, 2.81)] were lower in the DED group than in the control group [3.70 (2.54, 4.89)], with all differences reaching statistical significance (all *p* < 0.05).

**Table 4:** The concentration of amino acids in tear fluid.

Variable (μmol/L)	Total	DED with ocular pain (*n* = 15)	DED (*n* = 19)	Control (*n* = 16)	*F*/*H*	*p*	*q*-value
HIS	3.19 (1.32, 8.06)	3.15 (1.56, 15.23)	3.23 (0.75, 10.54)	2.90 (1.39, 6.28)	*H* = 1.050	0.591#	1.143
OHPRO	0.06 (0.03, 0.10)	0.08 (0.04, 0.15)	0.04 (0.02, 0.07)	0.08 (0.05, 0.14)	*H* = 5.529	0.063#	0.457
ARG	5.79 (2.53, 13.52)	6.24 (2.53, 19.43)	4.60 (0.76, 12.65)	6.80 (3.86, 14.04)	*H* = 1.147	0.563#	1.166
ASN	1.11 (0.53, 2.94)	0.87 (0.53, 5.15)	1.02 (0.23, 3.77)	1.45 (0.73, 2.65)	*H* = 0.752	0.686#	1.047
PHETH	0.09 (0.08, 0.26)	0.13 (0.08, 0.26)	0.13 (0.08, 0.29)	0.08 (0.07, 0.33)	*H* = 4.621	0.099#	0.479
TAU	9.31 (4.14, 16.95)	10.78 (3.95, 17.29)	5.78 (3.69, 11.66)	14.81 (6.85, 23.95)	*H* = 4.393	0.111#	0.460
GLN	0.39 (0.18, 1.03)	0.27 (0.07, 1.31)	0.39 (0.09, 1.03)	0.54 (0.37, 1.04)	*H* = 1.436	0.487#	1.412
SER	14.48 (4.42, 37.70)	13.34 (6.38, 77.08)	16.86 (2.17, 52.10)	13.56 (7.51, 31.91)	*H* = 0.351	0.839#	0.973
ETH	2.53 (1.19, 4.31)	2.59 (1.64, 4.34)	1.58 (1.07, 2.81)	3.70 (2.54, 4.89)^b^	*H* = 8.785	0.012#	0.116
GLY	8.55 (4.21, 25.35)	7.43 (4.25, 40.24)	9.97 (1.77, 30.95)	8.25 (5.43, 21.09)	*H* = 0.221	0.895#	0.895
ASP	2.31 (0.52, 6.96)	2.27 (0.95, 7.98)	2.36 (0.35, 8.09)	2.36 (0.93, 6.20)	*H* = 0.285	0.867#	0.931
CIT	1.81 (0.59, 6.82)	1.72 (0.74, 18.56)	1.85 (0.44, 4.79)	2.13 (0.65, 6.90)	*H* = 0.894	0.639#	1.090
GLU	2.69 (1.18, 7.46)	2.18 (1.36, 9.00)	2.66 (0.73, 6.65)	3.40 (1.89, 7.42)	*H* = 1.236	0.539#	1.303
beta-ALA	0.12 (0.05, 0.29)	0.09 (0.03, 0.18)	0.12 (0.04, 0.26)	0.21 (0.09, 0.36)	*H* = 3.538	0.170#	0.616
THR	3.89 (1.31, 9.13)	2.87 (1.35, 18.98)	4.25 (0.38, 12.17)	3.51 (1.92, 8.92)	*H* = 0.241	0.886#	0.918
ALA	6.79 (3.21, 17.56)	5.78 (3.87, 32.07)	7.27 (1.28, 22.92)	7.45 (3.98, 15.59)	*H* = 0.782	0.676#	1.089
gamma-GABA	0.02 (0.01, 0.03)	0.02 (0.01, 0.04)	0.02 (0.01, 0.03)	0.02 (0.02, 0.04)	*H* = 0.906	0.635#	1.151
OH-LYS	0.14 (0.11, 0.21)	0.19 (0.14, 0.30)	0.10 (0.10, 0.11)^a^	0.18 (0.13, 0.21)^b^	*H* = 36.684	<0.001#	<0.000
AMADP	0.07 (0.05, 0.11)	0.08 (0.05, 0.14)	0.06 (0.05, 0.08)	0.09 (0.06, 0.12)	*H* = 5.266	0.071#	0.412
PRO	1.91 (0.55, 5.50)	1.58 (0.81, 9.54)	1.90 (0.19, 3.50)	2.26 (1.71, 6.09)	*H* = 2.079	0.353#	1.137
ORN	2.44 (1.08, 5.72)	2.37 (1.21, 9.65)	2.46 (0.48, 5.72)	2.42 (1.48, 8.32)	*H* = 0.658	0.719#	1.043
LYS	2.06 (0.66, 4.21)	1.41 (0.60, 6.97)	1.73 (0.15, 4.21)	2.89 (1.25, 3.81)	*H* = 1.202	0.548#	1.222
TYR	0.72 (0.25, 2.71)	0.51 (0.25, 4.24)	0.74 (0.12, 2.71)	0.76 (0.34, 2.07)	*H* = 0.302	0.859#	0.958
MET	0.54 ± 0.62	0.97 ± 0.92	0.34 ± 0.22^a^	0.36 ± 0.37^a^	*F* = 6.396	0.003	0.044
VAL	1.64 (0.62, 5.31)	1.21 (0.67, 7.91)	1.84 (0.18, 5.87)	1.71 (0.90, 4.20)	*H* = 0.636	0.727#	0.958
ILE	0.99 (0.32, 2.95)	0.92 (0.46, 4.57)	1.05 (0.13, 3.21)	1.01 (0.39, 2.48)	*H* = 0.555	0.757#	0.954
LEU	2.32 (0.79, 5.82)	2.58 (0.79, 7.11)	2.10 (0.50, 5.92)	2.40 (1.45, 4.66)	*H* = 0.477	0.787#	0.951
PHE	2.43 (0.84, 3.90)	2.45 (0.84, 4.84)	2.32 (0.66, 3.90)	2.55 (1.32, 3.48)	*H* = 0.644	0.724#	1.000
TRP	4.05 (1.83, 7.21)	5.48 (2.16, 10.79)	2.54 (1.16, 6.47)	4.24 (2.05, 7.64)	*H* = 1.311	0.519#	1.368

^#^The rank sum test; ^a^indicates that there was a statistically significant difference between the DED with ocular pain group after pairwise comparison; ^b^indicates that the difference was statistically significant compared to the DED group after pairwise comparison.

DED, dry eye disease; HIS, L-histidine; OHPRO, L-hydroxyproline; ARG, L-arginine; ASN, L-asparagine; PHETH, O-phosphoethanolamine; TAU, taurine; GLN, L-glutamine; SER, L-serine; ETH, ethanolamine; GLY, glycine; ASP, L-aspartic acid; CIT, L-citrulline; GLU, L-glutamic acid; beta-ALA, β-alanine; THR, L-threonine; ALA, L-alanine; gamma-GABA, γ-aminobutyric acid; OH-LYS, δ-D, L-hydroxylysine; AMADP, L-α-aminoadipic acid; PRO, L-proline; ORN, L-ornithine; LYS, L-lysine; TYR, L-tyrosine; MET, L-methionine; VAL, L-valine; ILE, L-isoleucine; LEU, L-leucine; PHE, L-phenylalanine; TRP, L-tryptophan; μmol/L, micromoles per liter.

After applying FDR correction, tear fluid MET also remained a significant biomarker after correction (*q*-value = 0.044), though with a more moderate effect size.

### 3.5 The detection results of amino acids in plasma

**Figure 3:**
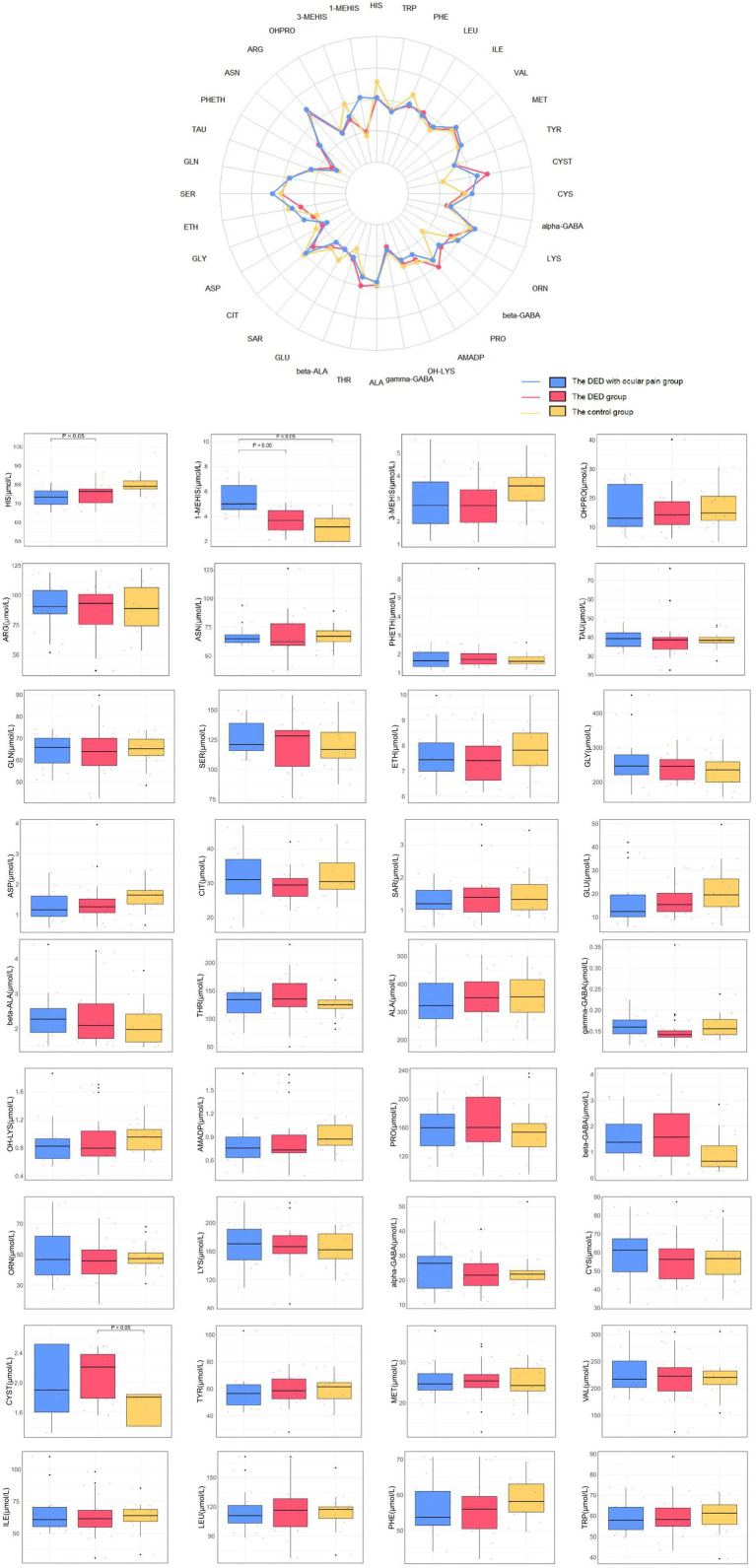
The differences in amino acid concentrations in plasma for the three groups. Top: Means values of 36 amino acid concentrations (μmol/L) of plasma of three groups are shown in the radar chart. Bottom: The average concentration of amino acids in plasma in three groups are shown in the box plots.

A total of 36 amino acids were detected in the plasma (Table 5; Figure 3). Significant differences were found in HIS (*H* = 9.504, *p* = 0.024), 1-MEHIS (*H* = 17.596, *p* < 0.001), and CYST (*H* = 9.504, *p* = 0.008) levels across the three groups. The 1-MEHIS concentration in the DED with ocular pain group [4.99 (4.46, 6.48)] was significantly higher than that in the other two groups [DED group: 3.66 (2.84, 4.47); control group: 3.15 (1.98, 3.90)], with all differences being statistically significant (all *p* < 0.05). Furthermore, HIS levels in the DED group (74.26 ± 7.77) were higher than those in the DED with ocular pain group (73.95 ± 6.02), and CYST levels [2.21 (1.78, 2.39)] were higher in the DED group than in the control group [1.81 (1.42, 1.84)], with all differences statistically significant (all *p* < 0.05).

**Table 5:** The concentration of amino acids in plasma.

Variable (μmol/L)	Total	DED with ocular pain (*n* = 15)	DED (*n* = 19)	Control (*n* = 16)	*F*/*H*	*p*	*q*-value
HIS	75.99 ± 7.20	73.95 ± 6.02	74.26 ± 7.77^a^	79.96 ± 6.18	*F* = 4.034	0.024	0.288
1-MEHIS	3.95 (2.95, 4.83)	4.99 (4.46, 6.48)	3.66 (2.84, 4.47)^a^	3.15 (1.98, 3.90)^a^	*H* = 17.596	<0.001^#^	<0.000
3-MEHIS	3.02 ± 1.09	2.89 ± 1.29	2.80 ± 0.97	3.39 ± 1.01	*F* = 1.412	0.254	1.143
OHPRO	14.18 (10.86, 20.18)	13.02 (9.98, 26.20)	14.21 (10.86, 18.85)	14.82 (11.96, 21.03)	*H* = 0.447	0.799^#^	1.199
ARG	88.29 ± 20.14	90.09 ± 19.32	86.87 ± 21.30	88.30 ± 20.66	*F* = 0.103	0.902	1.082
ASN	65.51 (60.43, 74.39)	64.89 (61.49, 69.51)	62.33 (58.45, 78.49)	67.17 (61.91, 72.25)	*H* = 0.070	0.965^#^	1.022
PHETH	1.64 (1.38, 2.00)	1.64 (1.32, 2.17)	1.71 (1.40, 2.04)	1.61 (1.43, 1.86)	*H* = 0.955	0.620^#^	1.240
TAU	38.64 (35.12, 40.43)	39.32 (35.12, 42.44)	38.55 (32.69, 40.03)	38.39 (36.95, 39.94)	*H* = 0.561	0.755^#^	1.182
GLN	64.40 ± 9.07	64.04 ± 7.51	64.51 ± 11.83	64.61 ± 6.92	*F* = 0.017	0.984	0.984
SER	122.19 ± 19.69	126.33 ± 15.08	120.21 ± 23.29	120.65 ± 19.45	*F* = 0.467	0.630	1.194
ETH	7.63 ± 1.03	7.70 ± 1.08	7.42 ± 0.97	7.82 ± 1.06	*F* = 0.698	0.503	1.207
GLY	245.85 ± 54.99	264.46 ± 75.41	242.34 ± 38.95	232.55 ± 46.90	*F* = 1.387	0.260	1.040
ASP	1.37 (1.11, 1.76)	1.16 (0.93, 1.64)	1.26 (1.03, 1.53)	1.65 (1.34, 1.80)	*H* = 4.411	0.110^#^	0.792
CIT	31.04 ± 6.81	31.67 ± 8.88	29.39 ± 4.83	32.42 ± 6.65	*F* = 0.949	0.394	1.091
SAR	1.31 (0.96, 1.71)	1.20 (0.98, 1.63)	1.39 (0.93, 1.71)	1.33 (1.00, 1.82)	*H* = 0.262	0.877^#^	1.128
GLU	15.69 (11.28, 23.84)	12.38 (9.98, 20.45)	15.34 (11.52, 20.28)	19.50 (14.11, 27.82)	*H* = 2.509	0.285^#^	1.026
beta-ALA	2.08 (1.63, 2.57)	2.27 (1.80, 2.58)	2.09 (1.63, 2.75)	1.98 (1.62, 2.44)	*H* = 1.491	0.474^#^	1.219
THR	131.21 ± 32.04	127.35 ± 24.33	140.29 ± 42.67	124.05 ± 21.02	*F* = 1.286	0.286	0.936
ALA	347.57 ± 87.22	341.18 ± 95.31	350.63 ± 89.50	349.92 ± 81.87	*F* = 0.055	0.946	1.032
gamma-GABA	0.15 (0.14, 0.17)	0.16 (0.14, 0.18)	0.14 (0.14, 0.16)	0.16 (0.14, 0.18)	*H* = 3.280	0.193^#^	1.158
OH-LYS	0.83 (0.71, 1.02)	0.82 (0.63, 0.95)	0.80 (0.67, 1.09)	0.95 (0.77, 1.08)	*H* = 1.935	0.380^#^	1.140
AMADP	0.82 (0.69, 0.97)	0.76 (0.61, 0.94)	0.73 (0.69, 0.97)	0.87 (0.78, 1.05)	*H* = 3.058	0.216^#^	1.111
PRO	160.04 ± 36.97	155.54 ± 30.34	166.11 ± 41.33	157.06 ± 38.48	*F* = 0.409	0.667	1.201
beta-GABA	1.45 ± 1.01	1.63 ± 0.94	1.73 ± 1.13	0.96 ± 0.76	*F* = 3.094	0.055	0.495
ORN	48.29 ± 13.81	50.57 ± 17.81	46.36 ± 13.76	48.46 ± 9.48	*F* = 0.381	0.685	1.174
LYS	167.23 ± 28.66	169.10 ± 30.69	168.36 ± 31.23	164.15 ± 24.85	*F* = 0.134	0.875	1.167
alpha-GABA	22.90 (18.21, 28.18)	26.90 (15.94, 30.19)	22.06 (17.36, 26.79)	22.49 (19.90, 24.01)	*H* = 0.370	0.831^#^	1.197
CYS	57.69 ± 13.78	60.16 ± 15.16	56.35 ± 12.88	56.97 ± 14.05	*F* = 0.344	0.711	1.163
CYST	1.84 (1.61, 2.35)	1.90 (1.60, 2.52)	2.21 (1.78, 2.39)	1.81 (1.42, 1.84)^b^	*H* = 9.504	0.008^#^	0.144
TYR	59.61 (51.91, 64.64)	56.53 (47.93, 63.36)	58.53 (51.91, 67.82)	61.50 (52.72, 64.62)	*H* = 1.152	0.562^#^	1.190
MET	25.45 ± 4.49	25.82 ± 4.42	25.40 ± 5.01	25.15 ± 4.16	*F* = 0.083	0.920	1.068
VAL	222.13 ± 38.43	226.21 ± 35.87	221.78 ± 45.53	218.72 ± 33.27	*F* = 0.143	0.867	1.200
ILE	62.08 (56.23, 70.09)	60.81 (53.97, 70.78)	61.52 (53.69, 69.51)	63.92 (59.30, 69.65)	*H* = 0.141	0.931^#^	1.047
LEU	116.58 ± 23.46	116.14 ± 23.30	118.53 ± 28.00	114.67 ± 18.45	*F* = 0.117	0.890	1.105
PHE	57.15 ± 7.19	56.63 ± 7.90	56.15 ± 7.93	58.83 ± 5.51	*F* = 0.648	0.528	1.188
TRP	59.93 ± 8.80	59.54 ± 7.92	60.16 ± 10.35	60.02 ± 8.11	*F* = 0.021	0.979	1.007

^#^The rank sum test; ^a^indicates that there was a statistically significant difference between the DED with ocular pain group after pairwise comparison; ^b^indicates that the difference was statistically significant compared to the DED group after pairwise comparison.

DED, dry eye disease; HIS, L-histidine; 1-MEHIS, 1-methyl-L-histidine; OHPRO, L-hydroxyproline; ARG, L-arginine; ASN, L-asparagine; PHETH, O-phosphoethanolamine; TAU, taurine; GLN, L-glutamine; SER, L-serine; ETH, ethanolamine; GLY, glycine; ASP, L-aspartic acid; CIT, L-citrulline; SAR, L-sarcosine; GLU, L-glutamic acid; beta-ALA, β-alanine; THR, L-threonine; ALA, L-alanine; gamma-GABA, γ-aminobutyric acid; OH-LYS, δ-D, L-hydroxylysine; AMADP, L-α-aminoadipic acid; PRO, L-proline; beta-GABA, D, L-β-aminoisobutyric acid; ORN, L-ornithine; LYS, L-lysine; alpha-GABA, L-α-aminobutyric acid; CYS, L-cystine; CYST, cystathionine; TYR, L-tyrosine; MET, L-methionine; VAL, L-valine; ILE, L-isoleucine; LEU, L-leucine; PHE, L-phenylalanine; TRP, L-tryptophan; μmol/L, micromoles per liter.

After applying FDR correction, plasma 1-MEHIS continued to demonstrate a highly significant association with DED with ocular pain (*q*-value < 0.001), indicating superior diagnostic potential.

### 3.6 The correlation between MET in tear fluid, 1-MEHIS in plasma, other clinical variables, and the DED with ocular pain

The results of the correlation analysis (Table 6) showed that the MET in tear fluid exhibited negative correlations with CNFD (*r* = −0.430, *p* < 0.05), CNBD (*r* = −0.379, *p* < 0.05), CNFL (*r* = −0.399, *p* < 0.05), CTBD (*r* = −0.345, *p* < 0.05) and CNFA (*r* = −0.312, *p* < 0.05), while positively correlated with age (*r* = 0.301, *p* < 0.05). In plasma, 1-MEHIS was positively correlated with DED duration (*r* = 0.346, *p* < 0.05), OSDI score (*r* = 0.396, *p* < 0.05), and NRS score (*r* = 0.556, *p* < 0.05), while negatively correlated with Schirmer I test results (*r* = −0.302, *p* < 0.05). To mitigate potential gender bias, *t*-tests revealed no statistically significant differences in MET and 1-MEHIS levels between male and female participants (all *p* > 0.05).

**Table 6:** The correlation analysis results between MET in tear fluid, 1-MEHIS in plasma and other variables.

Variable	MET in tears	1-MEHIS in plasma
Basic information		
Sex	0.159	−0.050
Age (years)	0.301*	0.006
Duration of DED (months)	0.257	0.346*
Average sleep time per day (hours)	−0.041	−0.175
Ocular examination		
Visual acuity	0.060	−0.141
IOP (mmHg)	0.162	−0.179
FBUT (sec)	−0.112	−0.195
Schirmer I test (mm/5 min)	−0.233	−0.302*
NEI score (0–15 points)	−0.013	0.011
The OSDI and NRS scores		
OSDI score (points)	−0.005	0.396*
NRS score (0–10 points)	0.182	0.556*
IVCM		
CNFD (number/mm^2^)	−0.430*	−0.098
CNBD (number/mm^2^)	−0.379*	−0.064
CNFL (mm/mm^2^)	−0.399*	−0.159
CTBD (number/mm^2^)	−0.345*	−0.066
CNFA (mm/mm^2^)	−0.312*	−0.147
CNFW (mm/mm^2^)	−0.018	0.143
CNFracDim	−0.294	−0.081

*Indicates that there is a correlation, *p* < 0.05.

MET, L-methionine; 1-MEHIS, 1-methyl-L-histidine; DED, dry eye disease; IOP, intraocular pressure; FBUT, tear film breakup time; NEI score, the National Eye Institute; OSDI, the Ocular Surface Disease Index; NRS score, the Numeric Rating Scale; IVCM, laser *in vivo* confocal microscopy; CNFD, corneal nerve fiber density; CNBD, corneal nerve branch density; CNFL, corneal nerve fiber length; CTBD, corneal total branch density; CNFA, corneal nerve fiber area; CNFW, corneal nerve fiber width; CNFracDim, corneal nerve fiber fractal dimension; mmHg, millimeter of mercury; sec, seconds; mm, millimeter; min, minutes; mm^2^, square millimeter.

### 3.7 ROC curve analysis for MET in tear fluid and 1-MEHIS in plasma

**Figure 4:**
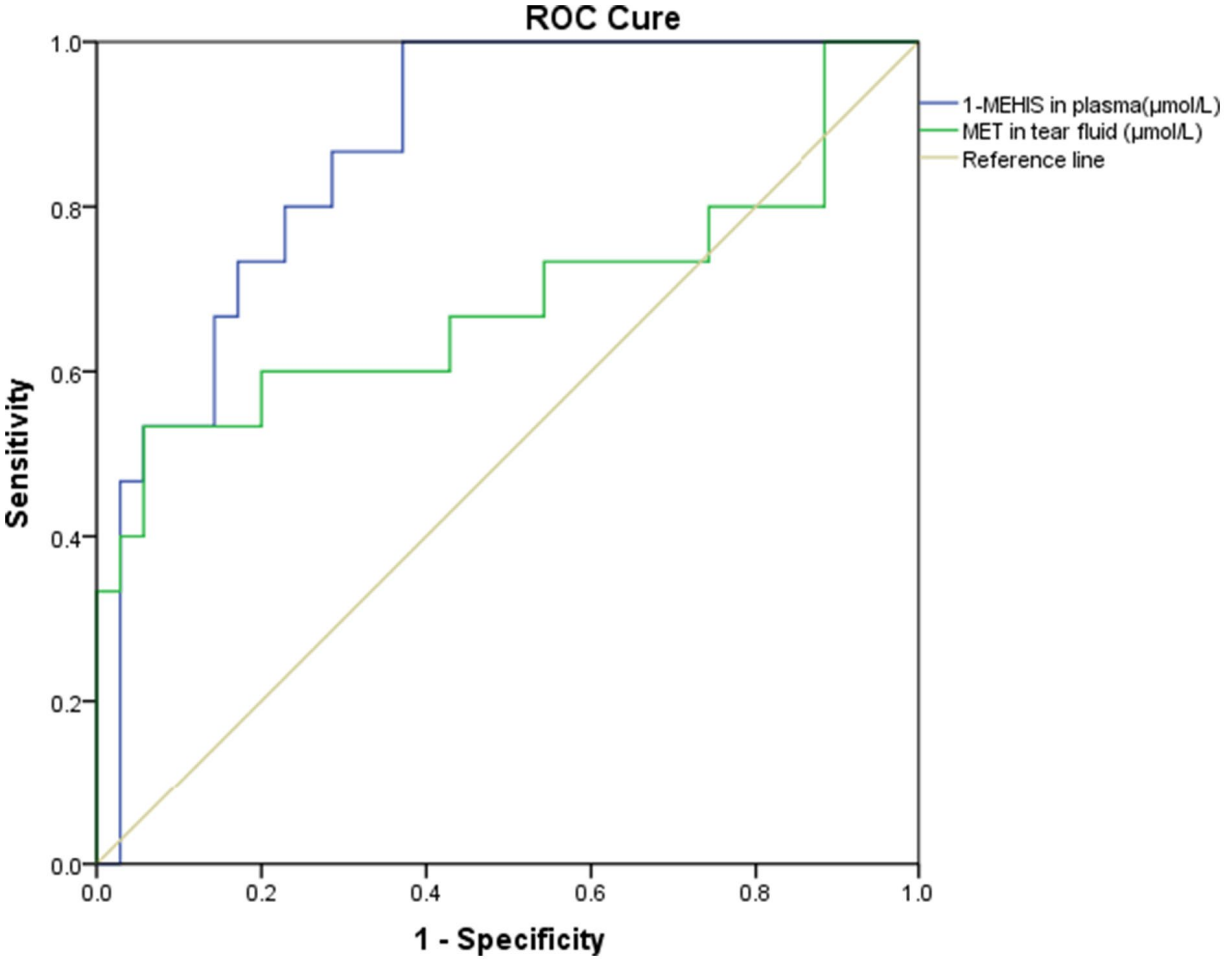
ROC curve for MET in tear fluid and 1-MEHIS in plasma in predicting DED with ocular pain (AUC: 0.686 and 0.869).

As MET in tear fluid and 1-MEHIS in plasma showed statistically significant differences among the three groups, ROC analyses were conducted (Figure 4). The area under the ROC curve (AUC) was 0.686 for MET and 0.869 for 1-MEHIS. The 95% confidence interval (CI) for the AUC of MET ranged from 0.495 to 0.877 (*p* = 0.039), whereas that of 1-MEHIS ranged from 0.770 to 0.967 (*p* < 0.001). Using the Youden index, the optimal cut-off value for MET was determined to be 0.770, yielding a sensitivity of 53.3% and a specificity of 94.3%. For 1-MEHIS, the optimal cut-off value was 3.779, achieving 100% sensitivity and 62.9% specificity.

## 4 Discussion

Metabolomics analysis of amino acids in tear fluid and plasma was conducted using ultra-performance liquid chromatography in this study. Notably, for the first time, significant elevations of MET in tears and 1-MEHIS in plasma were identified in patients with DED with ocular pain. After our rigorous statistical analysis, which included FDR correction, confirms 1-MEHIS in plasma as a robust biomarker candidate, showing remarkable resilience to multiple testing corrections. The significance of MET in tear fluid, while slightly attenuated after correction, still supports its potential role, albeit one that may require further investigation in larger cohorts to fully elucidate.

Amino acids are essential components for forming various ocular structures and maintaining their physiological functions. For example, collagen fibers in the corneal stroma, primarily composed of proline, lysine, glycine, and leucine, provide strength and transparency to the cornea (22). Abnormalities in amino acid metabolism or deficiencies in specific amino acids may contribute to ocular diseases (7, 8, 10, 23). Our study revealed that the concentrations of amino acids in tear fluid were lower than in plasma, a finding consistent with prior research (21). However, 29 amino acids were identified in tear fluid and 36 in plasma, surpassing previous studies in terms of the number of amino acids detected. This improvement can be attributed to the enhanced precision and sensitivity of modern mass spectrometry techniques, as well as a growing recognition of the link between amino acids and various diseases (21). Given that autologous serum eye drops closely resemble the physiological composition of human tears, they have been explored as a therapeutic option for DED (24, 25). Our results indicate that certain amino acids in plasma are present at much higher concentrations than in tear fluid, which may explain the clinical efficacy of plasma-based therapies for ocular surface disorders. The therapeutic effects may be attributed to the regenerative and anti-inflammatory properties of specific amino acids, which promote tissue repair and reduce inflammation (25, 26).

MET, or L-MET, is an essential amino acid vital for normal human growth and development. MET plays multiple critical roles in protein metabolism (27–31). One key function involves its conversion to S-adenosylmethionine (SAM) via the transmethylation reaction, with SAM serving as the principal methyl donor for most methylation reactions in the body. Disruption of this process can lead to homocysteine accumulation, which impaired ganglion cell apoptosis, reduced lysyl oxidase activity, and increased oxidative stress (30, 31). In addition, MET participates in the production of L-cysteine through transsulfuration. Cysteine acts as the rate-limiting substrate for GSH synthesis, combining with glutamate and glycine to form the tripeptide GSH. Dysfunction of the antioxidant pathway regulated by glutathione can lead to oxidative stress, exacerbating inflammatory responses and contributing to the progression of DED (32, 33). Studies have also demonstrated that administering MET at twice the recommended dietary dose for seven consecutive days in adult mice enhances microglial activation and inflammation in the brain while reducing hippocampal neurogenesis (34).

In our study, statistical differences were observed in CNFD, CNBD, CNFL and CTBD, particularly between the DED with ocular pain group and the control group. Our results also suggest that patients with DED with ocular pain may have a certain dissociation between symptoms (NRS) and signs (NEI score), which is consistent with neuropathic features and may indicate non-noceptive (e.g., neuropathic) mechanisms. These findings are consistent with previous studies (35, 36). In addition, our correlation analysis revealed that MET in tears is particularly negatively correlated with CNFD, CNBD, CNFL, CTBD and CNFA. These findings suggest that elevated MET levels in tears may disrupt amino acid metabolism in the ocular surface, contributing to corneal nerve damage and a reduction in corneal nerve density, fiber length, and area, which may ultimately lead to ocular pain. ROC curve analysis further supported its clinical relevance, yielding an AUC of 0.686, with an optimal cut-off value of 0.770, demonstrating 53.3% sensitivity and 94.3% specificity. Despite limited sensitivity, high concentrations of MET in tears are a highly specific indicator of DED with ocular pain. When elevated concentrations are detected, they strongly suggest the presence of a pathologic condition called ocular pain. This suggests that MET may be involved in specific biological pathways involved in the development of ocular pain. The underlying mechanisms of amino acid regulation in the tears of patients with DED with ocular pain remain poorly understood. Detailed information on the mechanisms of amino acid transport in corneal epithelial cells or tears is lacking in patients with DED or ocular pain. Thus, further experimental studies are needed to elucidate the role of MET in tear fluid in the pathophysiology of DED with ocular pain. Besides, the collection of tears for MET detection is more convenient and straightforward compared to IVCM examination, which has drawbacks such as the elevated requirement of patient cooperation and long duration. It can play a surveillance role in assessing changes in corneal nerves in patients with DED with ocular pain patients, and whether the disease progresses from DED to DED with ocular pain or even DED with ocular neuropathic pain.

In plasma, the content of 1-MEHIS in the DED with ocular pain group was significantly higher than in the other two groups. 1-MEHIS, a component of the dipeptide anserine (*β*-alanyl-N1-methyl-histidine), is primarily synthesized in animal muscle tissue, which explains its detection in plasma but not in tear fluid. Research on 1-MEHIS is predominantly focused on nutrition, with existing studies confirming a positive correlation between plasma levels of 1-MEHIS and the intake of animal proteins such as meat and fish (37, 38). In our study, ROC curve analysis of 1-MEHIS yielded an AUC of 0.877, with 100% sensitivity and 62.9% specificity, indicating a significant association between 1-MEHIS and DED with ocular pain. The high sensitivity and relatively low specificity observed may be attributed to its biological susceptibility to variations in dietary protein intake. Due to the lack of data on participants’ recent dietary intake of meat or fish, it is unclear whether the increased 1-MEHIS levels in plasma are related to recent animal protein consumption or the disease itself. Given that DED is a chronic condition with pathogenesis closely linked to diet, previous studies have shown that diets like the Mediterranean diet, characterized by lower meat consumption, can help mitigate DED (39, 40). Our results also suggest that plasma 1-MEHIS levels and NRS score influence each other, and the reason may be that ocular pain caused by chronic DED and its severity may be related to long-term high animal protein intake. Although the detection of plasma 1-MEHIS is an invasive procedure with certain limitations for clinical application, its 100% diagnostic sensitivity warrants further evaluation in prospective studies under strictly controlled dietary conditions.

Notably, MET in tear fluid and 1-MEHIS in plasma demonstrate complementary diagnostic performance. This observation suggests the potential development of a two-step biomarker strategy: first, using the high sensitivity of 1-MEHIS for initial screening to minimize false-negative results, followed by the high specificity of MET to confirm positive cases and reduce false-positive outcomes. Such a combined approach may significantly improve overall diagnostic accuracy compared to the use of a single biomarker.

This study has several limitations. First, although this study provides the most comprehensive amino acid profiling to date for patients with DED with ocular pain, the small sample size limits its conclusions and renders it exploratory in nature. Secondly, the Cochet-Bonnet or noncontact air-jet esthesiometry, which is not yet widely available in China, was not used in this study, preventing the assessment of the correlation between corneal sensitivity and amino acid concentrations. Thirdly, our study was not conducted in a Controlled Environment Laboratory (CELab), potentially introducing variability in amino acid detection.

In summary, our study has expanded the amino acid profiles in the tear fluid and plasma of patients with DED with ocular pain. A preliminary observation revealed a significant elevation in levels of MET in tears and 1-MEHIS in plasma, which were correlated with changes in corneal nerve morphology and NRS score, respectively, serving as possible biomarkers for DED with ocular pain. Future large-scale studies are still required to validate this conclusion.

## Data Availability

The raw data supporting the conclusions of this article will be made available by the authors, without undue reservation.
