# Supplementary material for: Metabolomic analysis identifies alterations of amino acids in the tears and plasma of patients with dry eye disease with ocular pain
Source: Front Med (Lausanne). 2026 Jan 9;12:1733856. doi: 10.3389/fmed.2025.1733856 (PMC12827585; doi:10.3389/fmed.2025.1733856)
Supplement: Supplementary file 1 [file Data_Sheet_1.pdf]

Supplementary Material

Appendix 1

Processing of Plasma and Tear Samples:

(1) The sample was thawed at room temperature and vortexed for 10 seconds to ensure uniformity.

(2) A 10 μL aliquot of plasma or 20 μL of tear fluid was transferred into a 1.5 mL centrifuge tube (Axygen MCT-150-C, Corning Incorporated). To this, 10 μL of ultrapure water, 5 μL of internal standard, and 40 μL of isopropanol containing 0.1% formic acid were added. The mixture was vortexed for 2 minutes.

(3) The centrifuge tube was placed in a desktop high-speed refrigerated centrifuge (Mikro 220R, Hettich Lab Technology) and centrifuged at 12,000 rpm at 4 °C for 10 minutes.

(4) After centrifugation, 10 μL of the supernatant was carefully transferred into a new 1.5 mL centrifuge tube, followed by the addition of 70 μL of borate buffer solution. The mixture was vortexed for 1 minute.

(5) To initiate derivatization, 20 μL of AccQ Tag derivatization reagent (Kairos amino acid kit, USA) was added, and the tube was shaken immediately for 10 seconds.

(6) After 1 minute, an excess of derivatization agent was added for hydrolysis. Once the derivatization reaction was complete, the centrifuge tube was placed in a 55°C water bath and heated for 10 minutes.

(7) After the reaction, 400 μL of ultrapure water was added to the solution, and the tube was vortexed for 1 minute to ensure thorough mixing.

(8) The sample was centrifuged at 12,000 rpm at 4 °C for 5 minutes in the same refrigerated centrifuge.

(9) After centrifugation, 150 μL of the supernatant was transferred into a liquid chromatography vial (5320, Dikma Technologies Inc.) for UPLC analysis.

Processing of Standards:

(1) Amino acid standard solutions were prepared through gradient dilution, yielding concentrations of 400, 200, 100, 40, 20, 10, 4, 2, and 1 μmol/L.

(2) For each standard solution, 20 μL of the solution, 5 μL of internal standard, and 40 μL of isopropanol (0.1% formic acid) were combined and vortexed for 2 minutes to ensure proper mixing.

(3-6) Steps 3 through 6 were followed as described in the processing of the plasma and tear samples.

(7) After adding 400 μL of ultrapure water for dilution, 100 μL of the supernatant was taken and transferred to a liquid chromatography vial for detection.

Table 1. Elution gradient

**Time（min）**	**Mobile phase**	
	**A（v%）**	**B（v%）**
0	96	4
0.5	96	4
2.5	90	10
5	72	28
6	5	95
7	5	95
7.1	96	4
9	96	4
